# Differences in physical aging measured by walking speed: evidence from the English Longitudinal Study of Ageing

**DOI:** 10.1186/s12877-016-0201-x

**Published:** 2016-01-28

**Authors:** Daniela Weber

**Affiliations:** Wittgenstein Centre (IIASA, ÖAW, WU), International Institute for Applied Systems Analysis, Schlossplatz 1, Laxenburg, 2361 Austria

**Keywords:** Walking speed, Population aging, Timed walk, ELSA

## Abstract

**Background:**

Physical functioning and mobility of older populations are of increasing interest when populations are aging. Lower body functioning such as walking is a fundamental part of many actions in daily life. Limitations in mobility threaten independent living as well as quality of life in old age. In this study we examine differences in physical aging and convert those differences into the everyday measure of single years of age.

**Methods:**

We use the English Longitudinal Study of Ageing, which was collected biennially between 2002 and 2012. Data on physical performance, health as well as information on economics and demographics of participants were collected. Lower body performance was assessed with two timed walks at normal pace each of 8 ft (2.4 m) of survey participants aged at least 60 years. We employed growth curve models to study differences in physical aging and followed the characteristic-based age approach to illustrate those differences in single years of age.

**Results:**

First, we examined walking speed of about 11,700 English individuals, and identified differences in aging trajectories by sex and other characteristics (e.g. education, occupation, regional wealth). Interestingly, higher educated and non-manual workers outperformed their counterparts for both men and women. Moreover, we transformed the differences between subpopulations into single years of age to demonstrate the magnitude of those gaps, which appear particularly high at early older ages.

**Conclusions:**

This paper expands research on aging and physical performance. In conclusion, higher education provides an advantage in walking of up to 15 years for men and 10 years for women. Thus, enhancements in higher education have the potential to ensure better mobility and independent living in old age for a longer period.

## Background

Physical and cognitive abilities vary with age across populations and population subgroups [1–4]. Walking is a measure of physical function which is related to musculoskeletal strength and power [5, 6] and is vital for independent life at higher ages [7]. There are several dimensions of walking such as gait rate, stride length, and walking speed (also termed gait speed). The measure walking speed is very popular in aging surveys as it can easily be assessed by nonprofessional staff [8].

Previous research has shown that walking speed declines with age and that men outperform women at all ages [9–11]. In a meta-analysis Bohannon *et al* identified walking speed to be relatively consistent between the age decades up to late 60s in both sexes; thereafter the mean pace was significantly less than in the previous decade [9].

Moreover, walking speed is associated with survival at all ages in both sexes, and is particularly informative for people aged 75 and over [12,13]. Physical capability measures such as walking speeds are also predictors of general health in the elderly [13]. This association can be explained by fact that individuals with higher walking speed also generally exhibit more healthy behaviors, lower cardiovascular risk, and lower levels of inflammatory markers [14].

The aim of this study is to investigate physical aging and particularly, the *walking age* of older adults in England. Life-expectancy varies by region and socioeconomic status, but is this also true for lower body functioning such as walking? What does your walking say about your true age? We convert the differences in walking speed (measured in m/s) between subpopulations into years of age.

## Methods

### Data source

We used the English Longitudinal Study of Ageing (ELSA), which is a longitudinal study of a representative sample of the English non-institutionalized population aged 50 years and older [15]. The first wave was collected in 2002 and thereafter participants were reinterviewed every second year until 2012. New samples were added in waves three, four and six [16,17]. Data were collected via face-to-face interviews using computer-assisted personal interviews and a self-completion questionnaire. In addition, a nurse visited participants in waves two, four, and six to measure physical functioning and to take blood samples and anthropometric measurements. Ethical approval for all the ELSA waves was granted by the National Research and Ethics Committee. All participants signed full informed consent to participate in the study. More information on ELSA can be found at http://www.ifs.org.uk/elsa/documentation.php.

In ELSA the response rates varied across the waves with 67 % in wave 1, 82 % in wave 2, 73 % in wave 3, 74 % in wave 4, 80 % in wave 5 and 81 % in wave 6 [17]. Details on conditional response rates are provided within the wave specific technical reports [16,17]. A recent study by Banks et al. [18] showed that there is no indication of a socioeconomic status (e.g education, income, and wealth) bias in attrition for participants aged at least 65 in ELSA, however, less-educated 55–64 year olds are more likely to drop out of the survey. For the present study we use all six available waves which provide us with panel data over a period of ten years. ELSA data are publicly available at http://discover.ukdataservice.ac.uk.

### Subjects

ELSA used a multistage, clustered, stratified sampling strategy. In total, about 17,980 participants were interviewed within all six waves. We restricted the data to participants aged between 60 and 89 years for this study and excluded people if their self-assessed age and the calculated age based on year of birth and survey year differed by more than two years. Only non-institutionalized older adults with complete data on walking speed were selected — a speed of less than 0.09 m/s was considered missing. We omitted data due to missing information for walking speed (12.9 %), height (15.3 %), weight (14.9 %), occupation (3 %), and education (8.1 %). These restrictions resulted in a final sample of 5,490 men and 6,221 women (N=11,711) and a total of 35,596 observations. Table 1 provides a descriptive overview for each wave.

**Table 1:** Descriptive sample overview by survey wave, including summary statistics such as mean (SD) on walking speed, age, height, and weight and shares of women, highly educated, non-manual worker, living in a wealthy region

Variables	Wave 1	Wave 2	Wave 3	Wave 4	Wave 5	Wave 6
walking speed	0.85 (0.28)	0.85 (0.27)	0.84 (0.28)	0.86 (0.28)	0.88 (0.27)	0.88 (0.28)
age	70.6 (7.4)	70.6 (7.4)	70.5 (7.6)	70.0 (7.6)	70.1 (7.5)	70.1 (7.4)
height	1.64 (0.09)	1.64 (0.10)	1.65 (0.09)	1.65 (0.09)	1.66 (0.09)	1.65 (0.10)
weight	74.5 (14.1)	75.1 (14.7)	75.9 (14.8)	76.8 (15.4)	77.8 (15.5)	77.6 (15.9)
females	54.47	54.85	53.94	53.57	52.72	52.97
higher educated	38.57	42.43	51.33	52.63	57.43	60.9
non-manual occupation	50.33	51.89	52.09	53.09	55.29	57.47
wealthier region	50.06	49.86	50.58	52.24	51.98	51.63
unweighted N	6107	5458	5181	6213	6330	6347

### Measures

#### Walking speed

Each participant aged 60 and above was eligible for the *timed walk* test. In addition, prior to the actual test respondents were asked if they had any problems from recent surgery, injury, or other health conditions that might prevent them from walking. Only persons aged at least 60 years, willing to do the test, and able to walk (walking aids were permitted) were asked to walk 8 feet (2.4 m) at their usual walking pace, twice. The time for both walks was recorded separately. In our analysis we use the mean speed (measured in m/s) of the two trials.

#### Anthropometrics

The anthropometric measures body weight and height were taken by nurses in waves 2, 4, and 6. A body weight of less than 29 kg and a height of less than 1.29 m were coded as missing information. In our analysis we included the mean height and weight of each participant to overcome the information lack in the odd waves.

#### Education

Information about the highest educational qualification was collected within the first interview. Thereafter (e.g. in the follow-up interviews) all participants were asked whether they had engaged in further education, and if so what. Participants could select one out of seven categories ranging from no qualification to higher education with degree. We recoded the seven categories into higher educated (NVQ2/GCE or higher) and lower educated (no qualification or NVQ1/CSE).

#### Occupation

Current or most recent job information was classified for each respondent using the National Statistics socio-economic classification. We dichotomized the variable by clustering managerial, professional, and intermediate occupations into non-manual occupation and routine and manual occupations into manual occupation.

#### Regional wealth

We used the Government Office Region information for each participant and categorized North East, North West, Yorkshire and the Humber, East Midlands, and West Midlands as the less wealthy region and East of England, London, South East, and South West as the wealthier region. A Regional Gross Value Added per head of less than £21,000 per capita in 2013 was used as cutoff point [19].

### Analysis

We identified differences in walking by subpopulations across age. The subpopulations were distinguished by three different characteristics (i.e. regional wealth and the two socio-economic classifications education and occupation), which are also associated with differences in life-expectancy.

Further, we explored the differences in physical functioning and aging with growth curve models [20,21]. This method allowed us to model changes in walking speed over time while not requiring observations at all waves for every participant. Moreover, we were able to consider individual heterogeneity in physical aging. The models were applied separately by sex and the three characteristics using a multilevel approach.

We identified a non-linear growth model with an age and age acceleration effect to properly represent the decline in walking speed, controlling for height, weight, and survey wave (verified by likelihood-ratio tests). We then added the dummy coded variable for the subpopulation with less education, manual occupation, and living in a less wealthy region as reference categories. Our analysis showed an effect of the subpopulation on the initial status, the between-person variability in walking speed. In addition, we included an acceleration subpopulation effect (i.e. an interaction of subpopulation and age^2^) for women and a slope subpopulation effect (i.e. an interaction of subpopulation and age) for men (all identified by likelihood-ratio tests).

The model for women was specified as follows:

$$\begin{array}{@{}rcl@{}} \begin{aligned} {}Y_{ti}&\! =\! \pi_{0i} \!+ \!\pi_{1i}({age}_{ti}\,-\,70) \,+\, \pi_{2i}{({age}_{ti}\,-\,70)}^{2} \,+\, \pi_{3i}{wave}_{ti}\,+\,e_{ti} \\ \pi_{0i}& = \!\beta_{00}+\beta_{01}{group}_{i}+ \beta_{02}{height}_{i} +\beta_{03}{weight}_{i} +r_{0i} \\ \pi_{1i}& = \!\beta_{10}+r_{1i} \\ \pi_{2i}& = \!\beta_{20}+\beta_{21}{group}_{i} \\ \pi_{3i}& = \!\beta_{30} \end{aligned} \end{array} $$

and for men

$$\begin{array}{@{}rcl@{}} \begin{aligned} {}Y_{ti}& \,=\, \pi_{0i} \,+\, \pi_{1i}({age}_{ti}\,-\,70)\! +\! \pi_{2i}{({age}_{ti}\,-\,70)}^{2} \,+\, \pi_{3i}{wave}_{ti}\,+\,e_{ti} \\ \pi_{0i}& =\! \beta_{00}+\beta_{01}{group}_{i}+ \beta_{02}{height}_{i} +\beta_{03}{weight}_{i} +r_{0i} \\ \pi_{1i}& = \!\beta_{10}+\beta_{11}{group}_{i}+r_{1i} \\ \pi_{2i}& = \!\beta_{20} \\ \pi_{3i}& = \!\beta_{30} \end{aligned} \end{array} $$

with *i* indicating the individual and *t* indicating the time. We assumed that the errors *e*_*ti*_ were independent and normally distributed with common variance *σ*^2^. In both models *π*_0*i*_ summarizes the influencing factors on the initial walking speed, *π*_1*i*_ represents the influences on the aging effect, and *π*_2*i*_ shows the influences on the age acceleration (age^2^). For readability issues, we centered age at 70 years, which was the mean age within our data sample. We also considered random variation between individuals’ initial walking status (indicated with *r*_0*i*_ within the model) and random variation of the age effect (indicated with *r*_1*i*_ within the model).

The magnitudes of the gap in physical aging due to education, occupation or regional wealth were converted into single years of age following a characteristic-based age approach [22,23]. To do this, walking speed was written as a function of chronological age and a set of covariates for each sex. These functions were used to calculate the so called *α*-ages *α*_*k*,*t*_, where *α*_*k*,*t*_ represents the chronological age of the subpopulation equivalent to a particular walking speed *k* at age *t* of the reference subpopulation [23]. Thus to highlight the differences in physical aging between subpopulations, we compare the ages of highly educated individuals, non-manual workers, and people living in wealthier regions against their counterparts who walk at the same speed.

We carried out all analyses and produced all figures using R 3.0.2 [24]. Sampling weights were used for all descriptive statistics to adjust for non-response and to ensure population representativeness. Sampling weights were not applied for the growth curve models because the longitudinal sampling weights provided by ELSA are only defined for participants who took part in all six waves. We preferred to include all new entrants regardless of whether they had missed one or more of the preceding waves.

## Results

In total our selected sample included 11,711 people who participated at least in one of the six waves of ELSA. About 12.7 % of the participants aged at least 60 had to be excluded, as they did not participate in the timed walking test. An overview of our sample including descriptive statistics by survey wave is presented in Table 1.

**Fig. 1:**
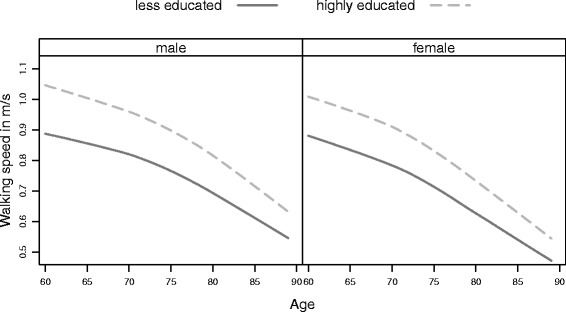
Men’s and Women’s age trajectories of walking speed by educational subpopulations. Average walking speed by age and subpopulation separately by sex showing the advantage of higher education in physical aging, particularly at younger ages

**Fig. 2:**
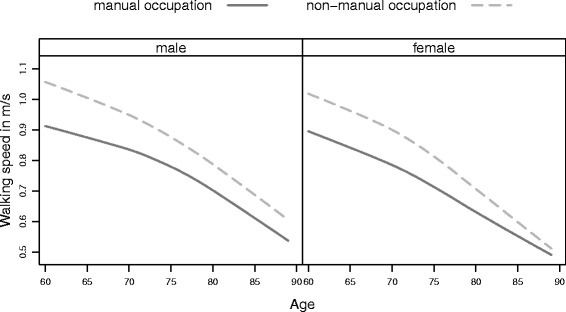
Men’s and Women’s age trajectories of walking speed by occupational subpopulations. Average walking speed by age and subpopulation separately by sex showing the advantage of non-manual occupation in physical aging, particularly at younger ages

**Fig. 3:**
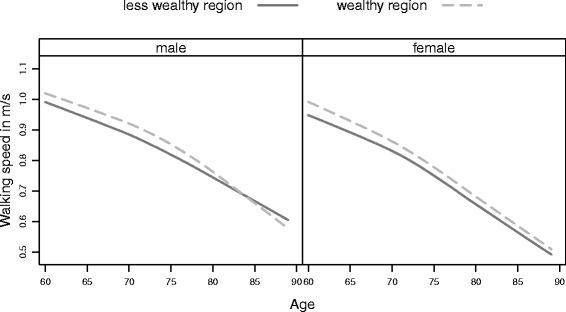
Men’s and Women’s age trajectories of walking speed by regional subpopulations. Average walking speed by age and subpopulation separately by sex showing a small advantage in physical aging when living in wealthier regions

As previously reported, men had faster walking speeds than women at all ages. However, differences were not only observed across sex, but also across subpopulations, demonstrating a socio-economic gradient indicating an advantage of higher socio-economic class for both sexes. Additionally, the descriptive analysis of the pooled six waves indicated a non-linear age effect on walking speed (Figs. 1, 2 and 3), which could be confirmed by growth curve models.

More highly educated men and women walked faster on average than their less educated counterparts. This difference was the greatest for men and women aged about 60 years (Fig. 1). More highly educated women aged 70 years walk on average 0.106 m/s faster than their less educated counterparts, whereas more highly educated men aged 70 years walk on average even 0.131 m/s faster than their less educated counterparts (Tables 2 and 3 Model 1). The age decline and the effect of age^2^ are highly significant for both men and women, but the acceleration effect is very small given that we have centered age at 70 years.

**Table 2:** Growth curve models for longitudinal changes in women’s walking speed addressing differences by education (Model 1), occupation (Model2), regional wealth (Model 3) and all three social factors (Model 4)

	Model 1	Model 2	Model 3	Model 4
	Estimate (SE)	Estimate (SE)	Estimate (SE)	Estimate (SE)
*Fixed Effects*				
Intercept	−0.100 (0.081)	−0.074 (0.077)	−0.182 (0.078) *	−0.023 (0.081)
Age	−0.013 (0.000) ***	−0.014 (0.000) ***	−0.014 (0.000) ***	−0.013 (0.000) ***
Age^2^	-0.0004 (0.000) ***	−0.0003 (0.000) ***	−0.0004 (0.000) ***	−0.0003 (0.000) ***
higher education	0.106 (0.007) ***			0.068 (0.008) ***
higher occupation		0.099 (0.007) ***		0.076 (0.008) ***
wealthier region			0.031 (0.007) ***	0.020 (0.007) **
Age^2^ x higher education	−0.0001 (0.000) *			0.0001 (0.000)
Age^2^ x higher occupation		−0.0002 (0.000) *		−0.0002 (0.000) *
Age^2^ x wealthier region			0.00002 (0.000)	0.0001 (0.000)
*Random Variance*				
Intercept	0.031 (0.18)	0.031 (0.18)	0.032 (0.18)	0.030 (0.18)
Linear Slope	0.0001 (0.01)	0.00007 (0.01)	0.00007 (0.01)	0.00007 (0.01)
Residual	0.023 (0.15)	0.023 (0.15)	0.023(0.15)	0.023 (0.15)
*AIC*:	−6145.3	−6823.3	−6697.6	−6164.6
*logLik*:	3088.7	3427.6	3364.8	3102.3

Note: Significance levels: * *p*≤0.05; ** *p*≤0.01; *** *p*≤0.001

Age was centered at 70 years; we control for height, weight and survey wave

Results indicate a decline of the educational advantage (Model 1) and occupational advantage (Model 2) with higher age; no significant regional wealth effect on individual aging; considering all three social factors only the decline of occupational advantage with higher age is significant

**Table 3:** Growth curve models for longitudinal changes in men’s walking speed addressing differences by education (Model 1), occupation (Model2), regional wealth (Model 3) and all three social factors (Model 4)

	Model 1	Model 2	Model 3	Model 4
	Estimate (SE)	Estimate (SE)	Estimate (SE)	Estimate (SE)
*Fixed Effects*				
Intercept	0.143 (0.091)	0.052 (0.089)	−0.073 (0.091)	0.210 (0.090) *
Age	−0.010 (0.001) ***	−0.010 (0.001) ***	−0.012 (0.001) ***	−0.009 (0.001) ***
Age^2^	−0.0004 (0.000) ***	−0.0004 (0.000) ***	−0.0004 (0.000) ***	−0.0004 (0.000) ***
higher education	0.131 (0.007) ***			0.102 (0.008) ***
higher occupation		0.105 (0.007) ***		0.067 (0.008) ***
wealthier region			0.025 (0.007) ***	0.007 (0.007)
Age x higher education	−0.0001 (0.001)			0.001 (0.001)
Age x higher occupation		−0.002 (0.001) **		−0.003 (0.001) **
Age x wealthier region			−0.0008 (0.001)	−0.001 (0.001)
*Random Variance*				
Intercept	0.032 (0.18)	0.033 (0.18)	0.035 (0.19)	0.031 (0.18)
Linear Slope	0.0001 (0.01)	0.0001 (0.01)	0.0001 (0.01)	0.00009 (0.01)
Residual	0.025 (0.16)	0.025 (0.16)	0.025 (0.16)	0.025 (0.15)
*AIC*:	−4620.1	−4733.4	−4504.1	−4718.5
*logLik*:	2326.1	2382.7	2268.0	2379.3

Note: Significance levels: * p ≤0.05; ** p ≤0.01; *** p ≤0.001

Age was centered at 70 years; we control for height, weight and survey wave

Results indicate a decline of the occupational advantage (Model 2) with higher age; no significant regional wealth effect on individual aging; considering all three social factors only the decline of occupational advantage with higher age is significant

The age and education interactions varied by sex. While women showed a significant interaction of education and age^2^ (Table 2 Model 1), the education-age interaction was not significant for men (Table 3 Model 1). Thus the advantage of higher education diminished at greater ages, in particular for women. Applying the *alpha*-age approach we showed that the difference in walking speed for men aged 60 can be converted into age years showing an educational disadvantage of 15.4 years representing a speed difference of 0.18 m/s (Table 4). Less educated women aged 60 had the same walking speed as their more educated counterparts who were almost 10 years older (Table 4). At older ages the gap reduced to 0.10 m/s for men and 0.06 m/s for women, which can also be read as an advantage of 5.1 age years and 2.6 age years respectively (Table 4).

**Table 4:** Women’s and men’s alpha-ages when being highly educated, a non-manual worker, or living in a wealthy region

	Women			Men		
Age	*α*-age	*α*-age	*α*-age	*α*-age	*α*-age	*α*-age
	education	occupation	region	education	occupation	region
60	70.8	69.2	64.1	75.4	73.1	65.1
65	73.4	72.4	67.8	76.7	74.4	68.0
70	76.5	75.9	72.1	79.1	76.8	71.9
75	80.0	79.6	76.7	82.3	80.0	76.2
80	83.7	83.4	81.4	86.1	83.7	80.8
85	87.6	87.2	86.2	90.1	87.7	85.5

In occupational subgroups, non-manual workers walked faster on average than manual workers (see Fig. 2). Interestingly, the gap in walking speed between the occupational subpopulations almost vanished at higher ages. In contrast to education, occupational subpopulations experienced significantly different aging in walking speed for both sexes, indicating a broad advantage of non-manual workers (Fig. 2, Tables 2 and 3 Model 2). Men showed a significant age and occupation interaction and women showed a significant occupation age^2^ interaction (Tables 2 and 3 Model 2). Hence, at an age of 60 years male manual workers performed at a 0.14 m/s lower level and 0.13 m/s for women, which can be also read as a disadvantage of 13 years for men and 9.2 years for women. Indicated by the significant interactions, the disadvantage was reduced to 2.2 years for women and 2.7 years for men, translating into 0.02 m/s and 0.08 m/s, respectively (Table 4).

There was a significant tendency for people living in wealthier regions to have slightly higher walking speeds (Fig. 3). Nevertheless, this gap was eliminated at higher ages. There was no significant interaction between age and regional wealth for either women or men (Tables 2 and 3 Model 3). The small regional differences were reflected in relatively small age differences of about four years at the age of 60 and one year at age 85 (Table 4).

After examining subpopulation differences in physical aging and determining the walking age of England’s older adults, we further explored the influence of the three social factors (education, occupational status, and regional wealth) on physical aging. The loglikelihoods and AICs of the growth models indicated that occupational status was more predictive than education or regional wealth for both men and women (Tables 2 and 3 Models 1–3). While the initial status of women’s walking speed was significantly associated with all three social factors only the occupation effect was significantly associated with the age^2^ aging effect (Table 2 Model 4). The results for men showed a similar pattern, where occupation was the only significant interaction with the aging effect. Regional wealth was not important for either men’s initial walking speed or the aging effect (Table 3 Model 4).

## Discussion

In this study we explored the non-linear age trajectory of walking speed as a proxy for physical aging of England’s older adults using six waves of the English Longitudinal Study of Ageing (ELSA). The mean walking speed declined by age for both sexes with men performing at higher levels than women across all ages. We also identified disparities in aging between subpopulations distinguished by socio-economic factors (education, wealth of residence, and whether occupation was manual or not). The advantage of higher education, non-manual work, or living in a wealthier region was shown to comprise up to 15 age years for men and up to 10 years for women. However, the magnitude of the advantage declined with increasing age to about 5 years for men and 2 for women.

Earlier research has highlighted the importance of walking speed, which is predictive of institutionalization, hospitalization, and mortality, particularly for older individuals [12,14,25,26]. Moreover, a loss of mobility represents a turning point in older person’s life as it has been linked with the “slowing down process” [8,11]. The decline in walking speed starts around age 60, previous research has found, and a speed of less than 0.6 m/s is associated with substantial impairments [8–10]. Older adults in particular should therefore aim to sustaining walking speed at a high level.

We found that higher levels of education were associated with a higher walking speed for both sexes and across all ages. A more highly educated 70 year old walked around 0.1 m/s faster at their normal pace than a less educated person of the same age, taking into account covariates. This was in accordance with previous studies [27,28]. For instance, in a cross-sectional study on older Swedish individuals higher education was associated with better lower extremity performance, such as walking speed, balance, chair stands, as well as upper extremity performance such as hand grip strength [27].

Studenski et al. reported an increase of 0.1 m/s in gait speed to be associated with a 12 % lower risk of death (hazard ratio of 0.88) [12]. An added value of our study is the conversion into *α*-ages, which translates the physical advantage into years of age. Roughly speaking, more highly educated 70 years old are associated with a 12 % lower risk of death than less educated of the same age, who walked 0.1 m/s slower. However, our findings suggested a faster acceleration of age decline among more highly educated women, but no significant interaction between education and age among men.

When looking at disparities in walking speed due to occupation, another dimension of socio-economic status, we received similar results. Older adults with higher occupational status performed at higher levels within the walking speed test than manual workers for both sexes across all ages. The advantage in walking speed due to higher occupational status was slightly less than the one due to higher education, which was also shown by *α*-ages. It strikes that there is a significant age occupational status interaction for both sexes, although we did not find a significant education age interaction for men.

Numerous studies have documented that higher socio-economic status is positively associated, for older individuals especially, with better physical functioning, such as upper extremity functions (e.g. hand grip strength) as well as lower extremity functions (e.g. walking speed, chair stand, and balance), but also with less impairments in daily activities [4, 27–29]. However, these studies were inconsistent when it came to the significance of the interaction between socio-economic status and age, which we could address by investigating separately the effect of education and occupation. Considering the wealth of the region of residence together with occupation and education, our results showed that occupation is more important for physical aging than education. This might be due to education often taking place at an early age, whereas occupation was more recent.

We also found that living in a wealthier region was positively associated with higher walking speeds. This was in accordance with previous studies showing a positive association between regional factors (e.g. GDP, unemployment rate, regional development) and well-being, as well as higher cognitive performance [3, 30]. However, the magnitude of the difference due to regional wealth was only minor in comparison to occupation status (i.e 0.03 m/s for men and 0.025 m/s for women). A recent study by van Cauwenberg et al. showed that older adults living in urban areas were more likely to walk daily for transportation than their counterparts living in semi-urban or rural areas [31]. This daily routine of walking for transportation instead of using the car might support the regional wealth effect on walking speed.

Some limitations of the study should be noted. First, there were some individuals dropped out of the longitudinal study, in particular core members after their first participation, and there was some missing data. Cross-sectional and longitudinal sampling weights are provided to account for nonresponse and national representativeness, however participants with nonresponse during some of the waves were not considered in the longitudinal weights. Missing data could bias our results if, for instance, less educated or manual workers are more likely to drop out. Banks et al. investigated attrition in ELSA and showed that for individuals aged at least 65 there is no significant relation between socio-economic status and attrition [18]. Second, the target population of ELSA were non-institutionalized individuals, although participants were followed when moving to institutions in the later waves. However, there were not many institutionalized participants, therefore people with severe physical and health limitations might be excluded.

In addition, non-mobile people were also excluded from our study. Participants might have chosen not to do the walking test due to lower body limitations or current sickness; we unfortunately do not know why participants chose not to participate. Therefore we might overestimate walking ability and underestimate the aging effect of the older adult population in England. Finally, we acknowledge that it is difficult to be sure that everyone walked at their normal pace and did not try to show their best walking performance.

Our analysis confirms the effects of education, occupational status, and regional wealth differences on physical functioning and furthermore highlights the differences by converting them into single years of age. Our research, along with previous studies, demonstrates that increasing equality could contribute to better physical functioning, which is critical to quality of life. Our results stress the need of policies to address socio-economic and regional inequalities in particular.

## Conclusions

This study identified differences in gait speed across socio-economic groups. Those in higher socio-economic groups had faster gait speed compared to lower socio-economic groups, although the size of the difference appeared to decline with advancing age. Greater understanding of this potential health inequality, highlighted by reporting the gait speed differences in years as well as meters per second, may enable better targeting of interventions to improve functional outcomes and independence in older age.

## Abbreviations

CSE: Certificate of secondary education
ELSA: English Longitudinal Study of Ageing
ft: feet
GCE: General certificate of education
GDP: Gross domestic product
m: meters
m/s: meters per second
NVQ1: National vocational qualification level 1
NVQ2: National vocational qualification level 2
SD: standard deviation
